# Why Fundamental Research Matters: Lessons from Nuclear Magnetic Resonance

**DOI:** 10.1021/acscentsci.6c00004

**Published:** 2026-03-10

**Authors:** Kirk S. Schanze

**Affiliations:** Department of Chemistry, 414492 University of Texas at San Antonio, San Antonio, Texas, 78249, United States

## Abstract

This In Focus article illustrates how curiosity-driven fundamental research in nuclear magnetic resonance (NMR) gave rise to transformative technologies, including modern spectroscopy and magnetic resonance imaging. By tracing this pathway from basic discoveries to broad societal and economic impact, the article serves as a resource emphasizing the essential role of sustained investment in fundamental scientific research.

## Graphical abstract

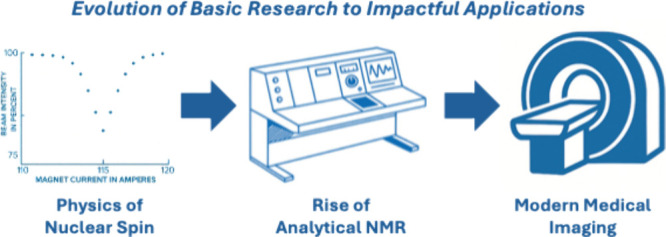

## Introduction and Intent

Over the past several years, it has become increasingly clear that scientists must better communicate the importance and broad societal benefits of curiosity-driven, basic scientific research. These benefits include the training of the next generation of STEM leaders and the advancement of human knowledge. Equally important, however, are the direct impacts of fundamental research on society through the development of new technologies that improve health, the general standard of living, and the economy. In this In Focus article, my goal is to highlight these impacts through the example of nuclear magnetic resonance (NMR) spectroscopy and imaging. This field emerged from basic research in physics and chemistry, whose long-term significance could not have been predicted when the basic discoveries were made. These early research breakthroughs, supported by both private and public funding, exemplify how investment in fundamental science can yield broad reaching and transformative applications. I have chosen NMR as a key example because, although I am not an expert in its development or implementation, throughout my career, my students and I have benefited enormously from the applications of NMR spectroscopy in our analyses of organic, organometallic, and inorganic materials. My early hands-on experience as an undergraduate student with a Varian A-60 spectrometer was an important factor in motivating me to pursue a career in chemistry. Finally, I note that this In Focus article is not intended as an authoritative review of the history of NMR or magnetic resonance imaging (MRI); readers seeking comprehensive reviews and primary references are directed to more specialized sources.^1^

## Nuclear Magnetic Resonance Spectroscopy

Several groups working on the basic physics of quantum effects arising from nuclear spin were responsible for the early discoveries that led to the development of NMR spectroscopy. These groups were aided by concurrent advances that occurred in the generation and measurement of radiofrequency (RF) and microwave electromagnetic radiation that was motivated among other things by the development of communications and radar during the second world war. The first important discovery comes from Rabi and co-workers who demonstrated that the deflection of a molecular beam of LiCl in a magnetic field could be induced by the application of a weak RF electromagnetic field (Figure 1).^2^ The beam deflection effect was attributed to the “resonance peaks of Li and Cl”. Rabi’s work was supported by the Ernst Kempton Adams Fund for Physical Research of Columbia University.

**Figure 1:**
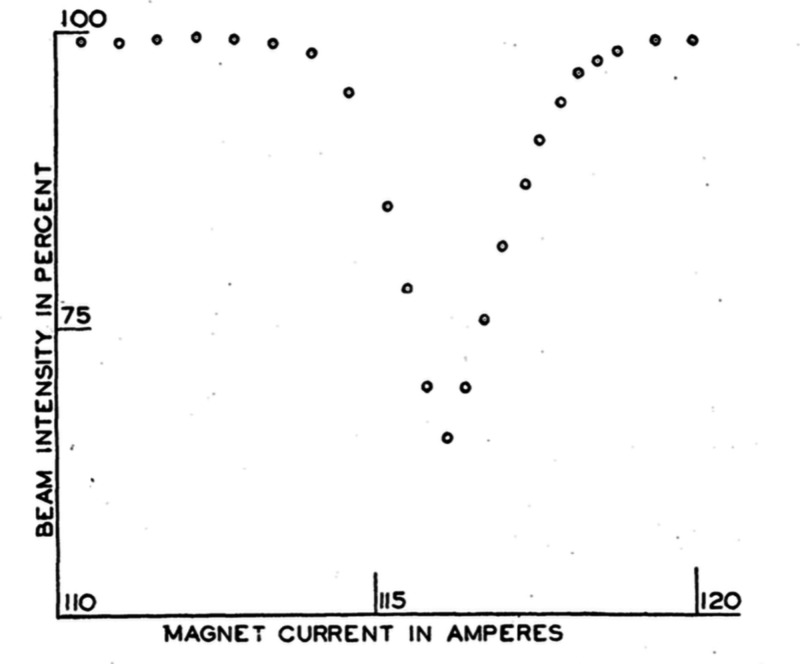
Deflection of a molecular beam of LiCl in a magnetic field by application of a weak radiofrequency field. Reproduced from ref 2. Copyright 1938 American Physical Society Journals.

Not long after Rabi’s work, two concurrent discoveries were made in which the effect of nuclear spin resonance was observed in condensed matter. First, Purcell and co-workers working at the Radiation Lab of the Massachusetts Institute of Technology (MIT) reported in a paper submitted on December 24, 1945, the absorption of RF by the protons contained in solid paraffin (hydrocarbon) that was placed in the cavity of a large electromagnet.^3^ Second, in a very short communication submitted a few weeks later, Bloch and co-workers reported observing proton resonance in a sample of water placed in a magnetic field.^4^ While the papers do not specifically indicate the source of support for the basic studies, Purcell was a Harvard Fellow on leave to the MIT Radiation Lab, which was supported by U.S. Office of Scientific Research and Development (OSRD). Rabi received the Nobel Prize in Physics in 1944 for the “resonance method for recording the magnetic properties of atomic nuclei”, and Purcell and Bloch shared the Nobel Prize in Physics in 1952 for “development of new methods for nuclear magnetic precision measurements”.

This field emerged from basic research in physics and chemistry, whose long-term significance could not have been predicted when the basic discoveries were made.

The following 15 years is a period where NMR development advanced rapidly, making it clear how the technique could be a powerful analytical tool for the chemical sciences. These developments were stimulated by the start-up company Varian Associates, which was the first occupant of the Stanford Research Park in 1953.^5^ Varian licensed the first patent on NMR and leveraged it to develop the first commercial NMR spectrometers (Figure 2a).^5^ These instruments allowed practicing scientists, who were not experts in electromagnets or RF engineering, to focus on the application of the spectroscopy to their scientific interests. Important discoveries include the chemical shift,^6,7^ spin–spin coupling,^8^ followed by applications of NMR to organic compounds of increasing complexity. The hallmark book written by J. D. Roberts (published in 1959)^9^ explained the fundamental principles and diverse applications of NMR to practicing organic chemists. Although detailed funding histories are difficult to reconstruct, published records show a combination of university, foundation, industry, and government funding supported the researchers who were advancing the field of NMR through their basic science investigations.

**Figure 2:**
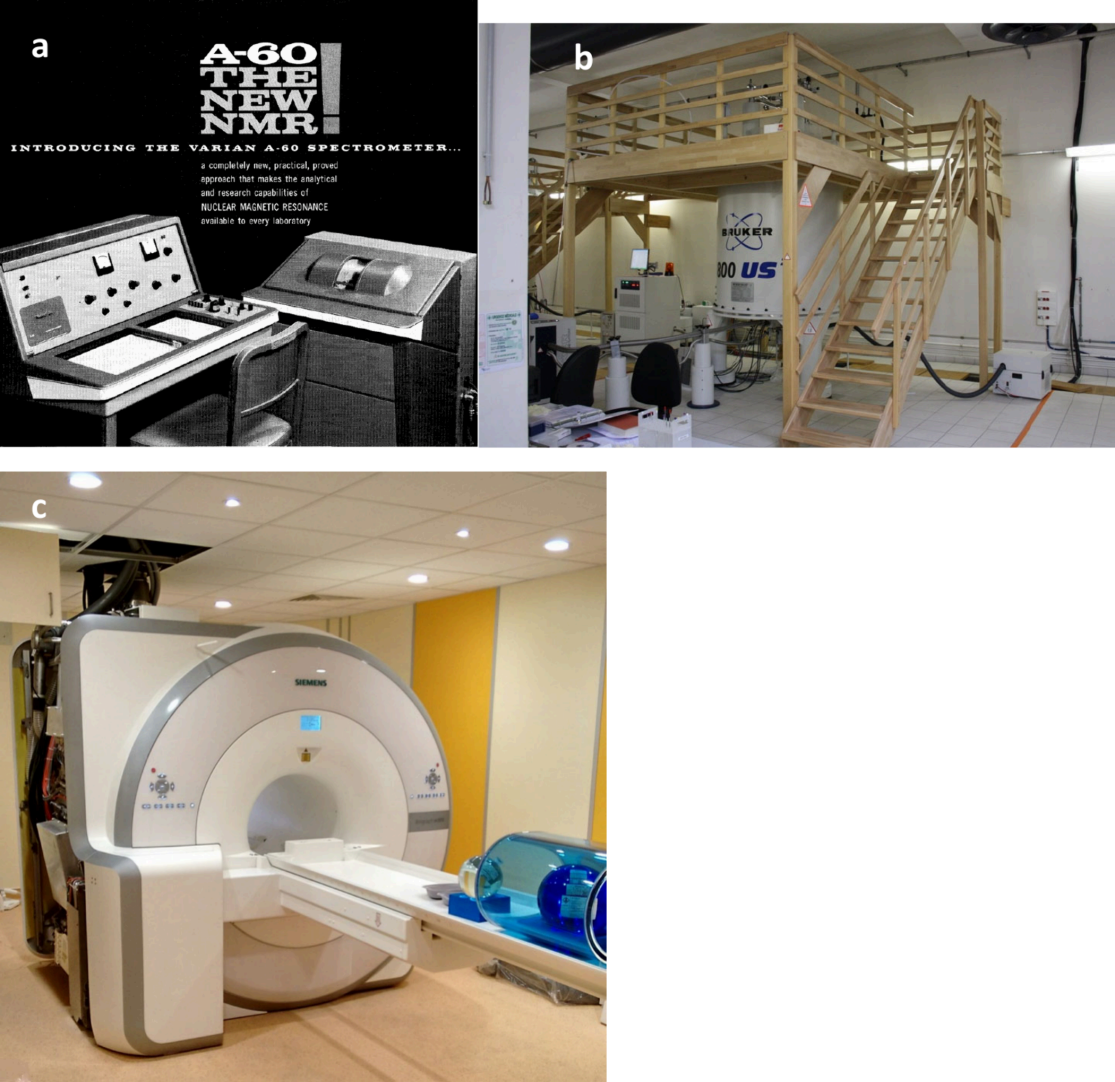
Advances in instrumentation have driven the scientific and medical applications of NMR. a) Advertisement for one of the first commercial NMR spectrometers, an electromagnet-based spectrometer that operated at 60 MHz for proton spectroscopy. Reproduced from ref 13. Copyright 1961 ACS Publications. b) Modern high field NMR spectrometer. Reproduced from ref 14. Available under a CC BY-SA 3.0 license. Copyright 2011 Lionel Allorge. c) MRI imaging machine. Reproduced from ref 15. Available under a CC BY-SA 4.0 license. Copyright 2015 Tomáš Vendiš.

During the decade between the late 1950s and late 1960s, the chemical applications of NMR advanced rapidly. Many chemistry departments gained access to NMR instruments allowing their routine use for analysis of organic and inorganic compounds and materials. While the use of NMR benefitted many subdisciplines of chemistry, probably the most immediate and profound effects were on the field of organic chemistry, where the use of routine proton NMR allowed rapid analysis of complex organic molecules, giving detailed insight into structural and stereoisomerism that was not available through other analytical methods. One example is the benefit to the structural elucidation of steroids, which were targets for synthetic drug development.^10^ While there are many implications of this scientific advance, it is immediately obvious that the field of drug development benefitted enormously, given that NMR allowed rapid advancements to synthetic chemical methods, organic structure identification including regio- and stereochemistry, and structure elucidation of natural products. The monetary and nonmonetary benefits of these advances are challenging to quantify, but their importance to society is clear.

The modern era of NMR spectroscopy began with the advent of pulsed Fourier transform (FT) NMR and the development of high field instruments based on cryogenic superconducting magnets (Figure 2b). The first practical demonstration of FT NMR was reported by Ernst and Anderson in 1967,^11^ but the widespread use of the technique was coupled to the development of minicomputers that could be deployed with an instrument allowing rapid, on-site processing of the data. The science and application of NMR continued to rapidly evolve through the 1970–1990 period, where a myriad of important applications were realized including the use of 2D NMR to elucidate the structure of proteins.^12^

With this perspective, one can readily appreciate the broad economic and societal contributions of NMR spectroscopy, both historically and today. Some of these benefits derive from the following areas: 1) Accelerated drug discovery and development; 2) chemical, food, agriculturals, and drug quality control and regulatory compliance; 3) increased efficiency in chemical and biochemical research and development; 4) acceleration in the development of polymers and materials that are important to many sectors including energy, electronics, aerospace, and petrochemicals.

All these benefits derive from the basic science studies that were described above, carried out by small groups of scientists that were supported by a variety of funding sources.

## Magnetic Resonance Imaging

The genesis of the application of nuclear magnetic resonance to biological imaging, magnetic resonance imaging (MRI), came from the contributions of several scientists who were motivated by an interest in basic discovery. Paul Lauterbur was a faculty member in a chemistry department when he devised the concept of using magnetic field gradients to produce 2D images from magnetic resonance signals. His work was first reported in 1973,^16^ and it was conducted using the Stony Brook University Chemistry Department’s Varian A-60 instrument.^17^ Soon thereafter, Peter Mansfield, a physicist at the University of Nottingham developed mathematical analysis techniques which allowed rapid imaging to be performed,^18^ giving rise to the practical method of magnetic resonance imaging. These early fundamental research breakthroughs catalyzed the rapid developments in the field which led to the first MRI images of humans,^19^ and commercialization of the technology in little more than a decade after the fundamental discoveries were made.

Although the early studies on MRI were done by small groups of scientists working with support from their institutions, MRI clearly represents one of the most important breakthroughs in medical science, arguably equaling the importance of X-ray imaging. Despite high capital and operational costs, MRI has become one of the most economically consequential technologies in modern medicine. The global MRI machine market exceeds $6–8 billion annually in equipment sales, and more if instrument support and service are included.^20^ Globally there are approximately 38,000 MRI scanners installed in hospitals and imaging centers (Figure 2c).^21^ The installed MRI instruments support tens of millions of diagnostic imaging studies each year, and each of these averages $1 thousand or more, amounting to a multibillion dollar medical industry that engages more than 100,000 medical professionals. In addition to direct clinical use, MRI also drives economic activity in biotechnology, pharmaceutical development, and medical AI, where imaging biomarkers and advanced reconstruction tools shorten clinical trials and enable new therapeutic strategies. As a result, MRI is both a major cost center and a major source of cost savings, forming a technology that has a strong influence on global healthcare spending and innovation.

Beyond the NMR to MRI trajectory highlighted in this article, ongoing advances in hyperpolarization, including parahydrogen based methods, dynamic nuclear polarization, together with multinuclear NMR (for example, ^13^C, ^15^N, ^31^P, ^129^Xe), are further expanding sensitivity, scope, and biomedical reach of magnetic resonance.^22–24^

Curiosity-driven, fundamental research can lead to unexpected and transformative breakthroughs.

## Summary

This In Focus article uses the example of nuclear magnetic resonance to illustrate how curiosity-driven, fundamental scientific research can lead to the development of novel, breakthrough technologies with far-reaching benefits for society and the global economy. It is a remarkable example showing that fundamental experiments conducted by a few scientists working independently can ultimately result in technologies with broad societal and economic impacts. I hope this example serves both as inspiration for young scientists and engineers pursuing innovative technologies and as a reminder to leaders in institutions and funding agencies who make decisions about resource allocation. While it may seem best to prioritize funding for developmental and applied research, it is essential to remember that curiosity-driven, fundamental research can lead to unexpected and transformative breakthroughs.

## Supplementary Material



## Notes

Transparent Peer Review report available (PDF)
