# Supplementary material for: Why Fundamental Research Matters: Lessons from Nuclear Magnetic Resonance
Source: ACS Cent Sci. 2026 Mar 10;12(4):391–5. doi: 10.1021/acscentsci.6c00004 (PMC13116816; doi:10.1021/acscentsci.6c00004)
Supplement: Supplementary file 1 [file oc6c00004_si_001.pdf]

oc-2026-00004b.R1

Name: Peer Review Information for "Why Fundamental Research Matters: Lessons from Nuclear Magnetic Resonance"

First Round of Reviewer Comments

Reviewer: 1

Comments to the Author

This format is relatively new for me, and there are not many of these contributions in ACS Central Science. They have very different types of content, and a historical survey of a relevant spectroscopy, such as NMR, is suitable for an In Focus article.

Having said that, I feel this short manuscript is only suited to a general audience, as most of its content is well known to the community.

I have appreciated the emphasis given to fundamental research and the need for dedicated funding schemes. I would have appreciated having an outlook on the challenges and potential of the technique. For instance, the shortage and high cost of helium are probably issues for less developed countries. What are the most promising technologies to solve this issue? Could discussing this issue stimulate and benefit from fundamental research in other fields?

On what goals is the NMR community working on?

Reviewer: 2

Comments to the Author

The In Focus manuscript illustrates most eloquently the need for basic research, for which the story of the NMR developments is an excellent choice.

It is very important to publish and promote such opinion pieces.

Science-wise, one wonders if it may be worth mentioning work on para-hydrogen, as well as dynamic nuclear polarization (DNP) and chemically induced dynamic nuclear polarization (CIDNP) (some of the above in the context of CERN), and the multinuclear NMR.

To reinforce the message for the general public, one could add the statistics on the growth of industries (monetary as well as the workforce) associated with for example MRI over the years, or how many people have received an MRI scan of some kind over the past decade.

Author's Response to Peer Review Comments:

Reviewer: 1

Recommendation: Major revisions required.

Comments:
This format is relatively new for me, and there are not many of these contributions in ACS Central Science. They have very different types of content, and a historical survey of a relevant spectroscopy, such as NMR, is suitable for an In Focus article.

Having said that, I feel this short manuscript is only suited to a general audience, as most of its content is well known to the community.

I have appreciated the emphasis given to fundamental research and the need for dedicated funding schemes. I would have appreciated having an outlook on the challenges and potential of the technique. For instance, the shortage and high cost of helium are probably issues for less developed countries. What are the most promising technologies to solve this issue? Could discussing this issue stimulate and benefit from fundamental research in other fields?

On what goals is the NMR community working on?

Response: We added a short paragraph that describes topics that are the focus of ongoing research in NMR and MRI, see response #1 to reviewer 2 below.

Reviewer: 2

Recommendation: Publish in ACS Central Science without change.

Comments:
The In Focus manuscript illustrates most eloquently the need for basic research, for which the story of the NMR developments is an excellent choice.

It is very important to publish and promote such opinion pieces.

Science-wise, one wonders if it may be worth mentioning work on para-hydrogen, as well as dynamic nuclear polarization (DNP) and chemically induced dynamic nuclear polarization (CIDNP) (some of the above in the context of CERN), and the multinuclear NMR.

To reinforce the message for the general public, one could add the statistics on the growth of industries (monetary as well as the workforce) associated with for example MRI over the years, or how many people have received an MRI scan of some kind over the past decade.

Response: To address the reviewer comments the following additions have been made:

1) A short paragraph was added to point to the new directions noted by the authors and three references to primary or review articles were added (refs. 20-22).

"Beyond the NMR to MRI trajectory highlighted in this article, ongoing advances in hyperpolarization, including parahydrogen based methods, dynamic nuclear polarization, together with multinuclear NMR (for example, ^{13}C, ^{15}N, ^{31}P, ^{129}Xe), are further expanding sensitivity, scope, and biomedical reach of magnetic resonance.[20-22]"

2) We added more statistics regarding the number of installed MRI scanners globally and an estimated number of MRI scans done annually.

"The global MRI machine market exceeds $6 - 8 billion annually in equipment sales, and more if instrument support and service are included.[18] Globally there are approximately 38,000 MRI scanners installed in hospitals and imaging centers.[19] The installed MRI instruments support tens of millions of diagnostic imaging studies each year, and each of these averages $1 thousand or more, amounting to a multi-billion dollar medical industry that engages more than 100,000 medical professionals."

oc-2026-00004b.R2

Name: Peer Review Information for "Why Fundamental Research Matters: Lessons from Nuclear Magnetic Resonance"

Second Round of Reviewer Comments

Reviewer: 1

Comments to the Author

The author has made minimal changes compared to prevous version.

I have no further comments.
